# Changes in TFG gene expression in bovine leucocytes transformed by *Theileria annulata*

**DOI:** 10.3389/fvets.2022.997294

**Published:** 2022-10-20

**Authors:** Hong-xi Zhao, Xia Li, Jun-long Liu, Gui-quan Guan, Jian-xun Luo

**Affiliations:** ^1^School of Agriculture, Ningxia University, Yinchuan, China; ^2^State Key Laboratory of Veterinary Etiological Biology, Key Laboratory of Veterinary Parasitology of Gansu Province, Lanzhou Veterinary Research Institute, Chinese Academy of Agricultural Sciences, Lanzhou, China

**Keywords:** tropomyosin-receptor kinase fused gene (TFG), *Theileria annulata*, immortalized, buparvaquone, polyclonal antibodies

## Abstract

*Theileria annulata* schizont-infected host cells in culture *in vitro* show unlimited proliferation similar to tumor cells; thus far, *T. annulata and T. parva* are the only eukaryotes that have been found to transform mammalian cells (immortalized). The transformation of these cells is reversible; when the parasite is eliminated in transformed cells by buparvaquone (BW720c), the host cells show normal growth and apoptosis. TFG is a tropomyosin-receptor kinase fused gene that is conserved among many species and is an important proto-oncogene. In this study, the bovine TFG gene was amplified by PCR from the cDNA of *T. annulata* schizont-transformed cells, cloned into the pGEX-4T-1 vector and expressed in *Escherichia coli* BL21 (DE3). After purification, the fusion protein was injected into rabbits to produce polyclonal antibodies. Using *T. annulata*-transformed cells together with BW720c treatment to kill the parasite, we aimed to identify changes in TFG gene expression by real-time PCR and Western blotting. The results showed that the bovine TFG gene was ~582 bp in size; SDS-PAGE analysis showed that the fusion protein was expressed in BL21 (DE3) cells with a molecular mass of 48 kD, and Western blotting indicated that the polyclonal antibodies could react with bovine TFG proteins from *T. annulata*-transformed cells and showed high specificity. Compared with that in the control group, the transcription level of the host TFG gene decreased significantly in the BW720c test group, and the expression of host tumor-related TFG protein decreased sharply after 72 h of drug treatment, suggesting that the TFG protein expression in transformed cells was directly related to *T. annulata*. This finding laid a foundation for further study on the interaction between *T. annulata* and host cells.

## Background

Bovine tropical theileriosis (*Theileria annulata* infection) is a tick-borne blood protozoonosis of cattle caused by infection of *T. annulata* (Apicomplexa, Piroplasmea, Theileriidae and *Theileria*). There are two stages (schizont and merozoite) of *T. annulata* in the host cells. Schizonts parasitize macrophages, dendritic cells and B lymphocytes, and merozoites parasitize red cells (1–3). Host cells infected with schizonts were cultured *in vitro*, and tumor-like cells with unlimited proliferation could be obtained (4). *T. annulata* and *T. parva* are the only known eukaryotes that can transform mammalian cells, and this kind of transformation induced by *T. annulata* is completely reversible (5). When the *Theileria* is killed by buparvaquone, *Theileria*-transformed cells will lose the uncontrolled proliferation and undergo apoptosis (6–8). *T. annulata* is considered an ideal model for better understanding the interactions between parasites and host cells.

The transformation of host cells is a complex biological process. Many studies have shown that *T. annulata* regulates cell phenotypes by acting on the cellular transcription factors and signaling pathways of host cells. In this process, many host cytokines and their signaling pathways, including NF-κB (nuclear factor kappa-B), MAPK (mitogen-activated protein kinase), PI3K (phosphoinositide 3-kinase), HIF1α (hypoxia-inducible factor-1α), JNK (c-Jun N-terminal kinase), PKM2 (pyruvate kinase M2), ubiquitin-like proteins ISG15, p53, c-Myc, and AP-1, were activated (9–18). The study found that *T. annulata* can recruit IKK signalosomes to its surface and phosphorylate to degradate IkB (inhibitor of NF-κB), which can induce NF-κB to enter the nucleus and inhibit apoptosis by increasing the expression of antiapoptotic proteins (c-FLIP, XIAP, and cIAP1/2) (16). In the p53-mediated signaling pathway, P53 protein was found to be mainly localized in the cytoplasm of host cells and closely linked with the schizont membrane. When buparvaquone (BW720c) was applied to transformed cells, the elimination of parasites led to the continuous entry of p53 into the host nucleus, upregulating the expression of the apoptotic precursor proteins Bax (BCL2-Associated X) and Apaf-1 (apoptotic protease activating factor-1) and downregulating the expression of the antiapoptotic protein Bcl-2, causing host cells to enter apoptosis (8, 13, 19). In the JNK signaling pathway, schizonts can activate JNK, induce c-Jun phosphorylation and activate the transcription factor AP-1 to regulate target gene transcription (11). Marsolier et al. also found that the parasite can participate in the regulation of the JNK signaling pathway through the interaction between TaPIN1-FBW7α and C-Jun (18). In addition, the TGF-β/Smad, ISG15, and Notch signaling pathways were found to be activated in *Theileria*-infected cells (17, 20, 21). However, how *T. annulata* interacts with these molecules, activates their signaling pathways and induces host cell transformation remains to be further studied.

TFG is a tropomyosin-receptor kinase fused gene that is conserved among many species and is an important proto-oncogene. Activated TFG protein can promote tumor development and regulate cell size, cell apoptosis and cell proliferation (22–25). In the early stage of infection, through the construction of a suppression subtractive library of transformed cells, the tumor-related gene TFG was screened, and we speculated that TFG was activated in transformed cells (26). Accordingly, we aimed to identify changes in TFG gene expression in bovine leucocytes transformed by *T. annulata*. First, in this study, the bovine TFG gene was amplified and expressed, and polyclonal antibody prepared. We found that homemade TFG antibodies could react with TFG proteins and show high specificity. Furthermore, we identified changes in TFG gene expression by real-time PCR and Western blotting. The results helped to lay the groundwork for further analysis of the molecular mechanisms of interactions between *T. annulata* and host cells.

## Methods

### *T. annulata* schizont-transformed cell line

*T. annulata* schizont-transformed cells lines were provided by the Vector and Vector-borne Disease (VVBD) laboratory, Lanzhou Veterinary Research Institute (LVRI), China. The cells were cultured using RPMI 1640 (Gibco, Grand Island, USA) culture flasks supplemented with 10% fetal bovine serum (Biological Industry, Kibbutz Beit Haemek, Israel), 100 U/mL penicillin (Sigma-Aldrich, St. Louis, MO, USA), and 100 mg/L streptomycin (Sigma-Aldrich). The cells were cultured in an incubator containing 5% CO_2_ at 37°C and passaged once every 2–3 days.

### Primer design and synthesis

Based on the bovine TFG gene sequence (GenBank gene no. BC104525.2), the prokaryotic expression primers of TFG (194) (TFG N-terminal: 1-194 amino acids) were designed by Primer Premier 5.0. BamHI and XhoI restriction sites and protective bases were introduced at the 5′ end of the sense and antisense primers. Sense primer: 5′-cgcggatccATGAATGGGCAGTTGGATCTGAGTG-3′ (BamHI); antisense primer: 5′-ccgctcgagTTATGAAACCTGATCATCTGTT-3′ (XhoI). The primers were synthesized by Shanghai Shenggong Bioengineering Technology Service Co., Ltd.

### Amplification of the TFG (194) gene

The TFG (194) gene was amplified with specific primers, and cDNA of the *T. annulata* schizont-transformed cell was used as a template. The DNA product was run in an agarose gel (1%). The target fragment was cut out, recovered, digested with BamHI and XhoI, and then linked with pGEX-4T-1 double enzyme cleavage products overnight. The ligation products were transformed into Trans 5α competent cells (TransGen Biotech, Beijing, China), and the positive clones were screened on LB plates containing ampicillin resistance. The positive clones were named pGEX-4T-1/TFG (194) after identification by PCR, double digestion and sequencing.

### Expression and purification of the pGEX-4T-1/TFG (194) protein

pGEX-4T-1/TFG (194)-positive plasmids were transformed into *E. coli* BL21(DE3), and a single colony was selected and inoculated in LB liquid medium containing 50 μg/mL ampicillin and cultured overnight at 37°C. The next day, the cultures were inoculated in the above liquid medium at a proportion of 1:100. When the conditions were 37°C and 180 rpm/min and the OD_600_ nm reached 0.6–0.8, IPTG was added to a final concentration of 1.0 mmol/L. The cultures were incubated successively for 4 h, the cells were centrifuged at 4°C and 12,000 rpm/min for 10 min, and finally, the bacterial precipitate was collected. Bacterial precipitation was resuspended in 10 mL of PBS. Ultrasound (100 W, 5 s ultrasound, 5 s intermission, 5 min in total) was carried out, and the ultrasonically crushed substance was centrifuged at 4°C and 12,000 rpm/min for 5 min to collect the precipitation. We washed the precipitate with 30 mL of inclusion body wash buffer 1 (50 mmol/L Tris-Cl, 10 mmol/L EDTA, 1% Triton-100, pH 8.0) and 30 mL of inclusion body wash buffer 2 (50 mmol/L Tris-Cl, 2 mol/L urea, pH 8.0) in turn. Finally, the precipitate was dissolved in phosphate buffer containing 8 mol/L urea, 2 × SDS-PAGE sample buffer was added, and the sample was heated in a water bath at 99°C for 8 min. Then, SDS-PAGE protein electrophoresis was performed. After electrophoresis was performed, staining was performed with Coomassie Brilliant Blue R250 and then decolorized with a decolorizing solution until protein bands appeared. The target protein was cut out, recovered and placed in a dialysis bag overnight, and the liquid in the dialysis bag was collected. Acetone was added at a 1:5 volume to precipitate the protein, and the protein was redissolved in protein diluent (25 mmol/L Tris, 0.1% SDS). The expression and purification of recombinant protein were analyzed by SDS-PAGE and Western blotting, respectively.

### Antibody preparation of the pGEX-4T-1/TFG (194) protein

Purified fusion protein (0.5 mL, ~500 μg) was mixed with Freund's complete adjuvant at a proportion of 1:1. After full emulsification, rabbits (~2.5 kg) were immunized with the subcutaneous multipoint method (permit number LVRIAEC-2018-001). Thereafter, immunization was performed in the same manner once every 2 weeks at 1/2 of the initial immunization dose mixed with 1:1 Freund's incomplete adjuvant to enhance immunization. After the last immunization for 10 days, blood was collected from the ear vein, and the antibody titer was determined by ELISAs. Briefly, 96-well microtiter plates (Nunc, Roskilde, Denmark) were coated with 100 μl/well of recombinant antigen at a concentration of 10 μg/ml in a coating buffer (0.1 M carbonate–bicarbonate buffer, pH 9.6). Ninety-six-well microtiter plates (Nunc, Roskilde, Denmark) were coated with 50 μl recombinant antigen at a concentration of 2.8 μg/ml in a coating buffer (0.1 M carbonate–bicarbonate buffer, pH 9.6) at 4°C overnight. The plates were washed three times with PBS-Tween (0.05% Tween 20; Merck, Darmstadt, Germany) and blocked with PBST (containing 5/100 skim milk powder) at 37°C for 1 h; then, the rabbit serum with different dilutions (1:200, 1:400, 1:800……1:25,600) was added as the primary antibody, followed by incubation at 37°C for 1 h and washing 3 times; The goat anti-rabbit IgG labeled with horseradish peroxidase (HRP) (Sigma, St. Louis, MO, USA) as the secondary antibody were added into each well, incubated the sample at 37°C for 1 h and washed 3 times; TMB (3,3′,5,5′-Tetramethylbenzidine; Sigma Aldrich, St Louis, MO, USA) was added for 10 min to color, and the reaction was terminated by adding termination liquid; finally the absorbance value of OD_450nm_ was read by an ELISA reader (microplate reader Model680, Bio-Rad, USA). To verify the ability of the polyclonal antibody to recognize the TFG protein from *T. annulata* transformed cells by Western blotting, the lysate of *T. annulata* schizont-transformed cells, bovine PBMCs (27) and *T. annulata* schizont was used as the antigen, respectively. Schizonts were purified as described by Abbasnia et al. (28). Briefly, *T. annulata* schizont-transformed cells were incubated with nocodazole to depolymerize microtubules. Then, Cells treat with trypsin-activated aerolysin on ice, and removing excess aerolysin with phosphate buffered saline. Host cell debris and nuclei were separated from schizonts of *T. annulata* by Percoll gradient centrifugation. Purified schizonts pellets were stored at −80°C. The primary antibody was immune rabbit serum, and the secondary antibody was HRP-conjugated donkey anti-rabbit IgG (Abcam, United States), proteins were detected by chemiluminescence (Thermo Fisher Scientific, United States), which can help us test the antibody quality.

### Real-time PCR and western blotting verification of TFG (194)

The test group and the control group were included in the experiment. *T. annulata* schizont-transformed cells were used as the test group, and PBMCs from uninfected bovine was used as the control group, with six replicates in each group. Twelve hours before drug treatment, the cell concentration was adjusted to 1 × 10^4^ cells/mL. First, BW720c (Sigma, St. Louis, MO, USA) prepared with DMSO (Sigma, St. Louis, MO, USA) was added to three replicates cells of *T. annulata* schizont-transformed cells and PBMCs, respectively. The final concentration of BW720c (the concentration of reserve solution was 1.5 mmol/L) was 200 ng/ml, and the same volume of DMSO was added to another three replicates cells, respectively. The cells were placed in the cell incubator for further culture and then sampled at 0, 12, 24, 36, 48, 60, 72, 84, and 96 h. The number of living cells at each time point was calculated by trypan blue staining, and the cell growth curves of the *T. annulata* transformed cells after drug treatment were plotted. At 12, 24, 48, 72, and 96 h, the cells were photographed by cell microscopy to observe their growth state. The cells were collected at 0, 24, 48, and 72 h and centrifuged at 2,000 rpm/min for 10 min. The supernatant was discarded, and the cell precipitation remained. The total RNA of the test group and control group was extracted by using RNeasy Mini kit (QIAGEN, Dusseldorf, Germany) based on the manufacturer's instruction. The first strand cDNA was synthesized by reverse transcription. We analyzed TFGs by real-time fluorescent quantitative PCR (sense primer TFG-F: TAGTTTGGAACCACCTGGAGAA; antisense primer TFG-R: CGCTTTTCTTCCCTACCATCCAC, the reference gene was bovine GAPDH, sense primer bGAPDH-F: GATGGTGAAGGTCGGAGTGAAC; antisense primer bGAPDH -R: GTCATTGATGGCGACGATGT). For the other part of the samples, Western blotting was used to verify the protein in the test group by using homemade anti-bovine TFG (194) protein antibody and a commercial mouse anti-β-actin (1/1000, Santa Cruz, United States) antibody.

### Data and statistical analyses

Data processing and graphics production were accomplished by using GraphPad Prism version 8. The results represent the mean ± standard error of three repeated independent experiments for all figures. Data were analyzed with the two tailed Student's *t*-tests. NS, not significant (*p* > 0.05), ^*^p < 0.05 and ^****^p < 0.0001.

## Results

### TFG (194) gene amplification and vector construction

**Figure 1:**
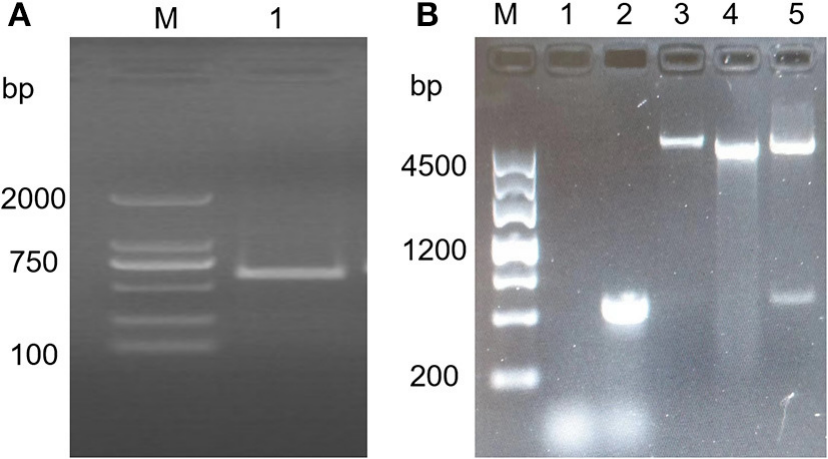
TFG (194) gene amplification and vector construction. **(A)** Amplification of TFG (194) gene by PCR. M: DL 2000 Marker; lane 1: The PCR amplification of TFG (194) gene. **(B)** Identification of recombinant plasmid pGEX-4T-1/TFG (194) by restriction enzyme digestation and PCR amplification. M:DL 4500 Marker; lane 1: Negative control; lane 2: The PCR-Amplificated of recombinant plasmid pGEX-4T-1/TFG (194); lane 3: Recombinant plasmid pGEX-4T-1/TFG (194); lane 4: pGEX-4T-1 plasmid; lane 5: Restriction enzyme identification result of recombinant plasmid pGEX-4T-1/TFG (194).

The TFG (194) gene was amplified by polymerase chain reaction (PCR), and the cDNA of *T. annulata* schizont-transformed cells was used as a template. The PCR product was detected by 1% agarose gel electrophoresis, and the amplified fragment size was ~582 bp (Figure 1A), which was consistent with the expected size. The target band was cut out, recovered, subjected to double enzyme digestion and then ligated with the pGEX-4T-1 double enzyme digestion product. The ligation product was transformed into Trans 5α competent cells, and positive clones were screened by LB plates containing ampicillin resistance. The positive clones were correctly identified by PCR, digestion and sequencing and were named pGEX-4T-1/TFG (194) (Figure 1B).

### Expression of the pGEX-4T-1/TFG (194) protein and preparation of polyclonal antibody

**Figure 2:**
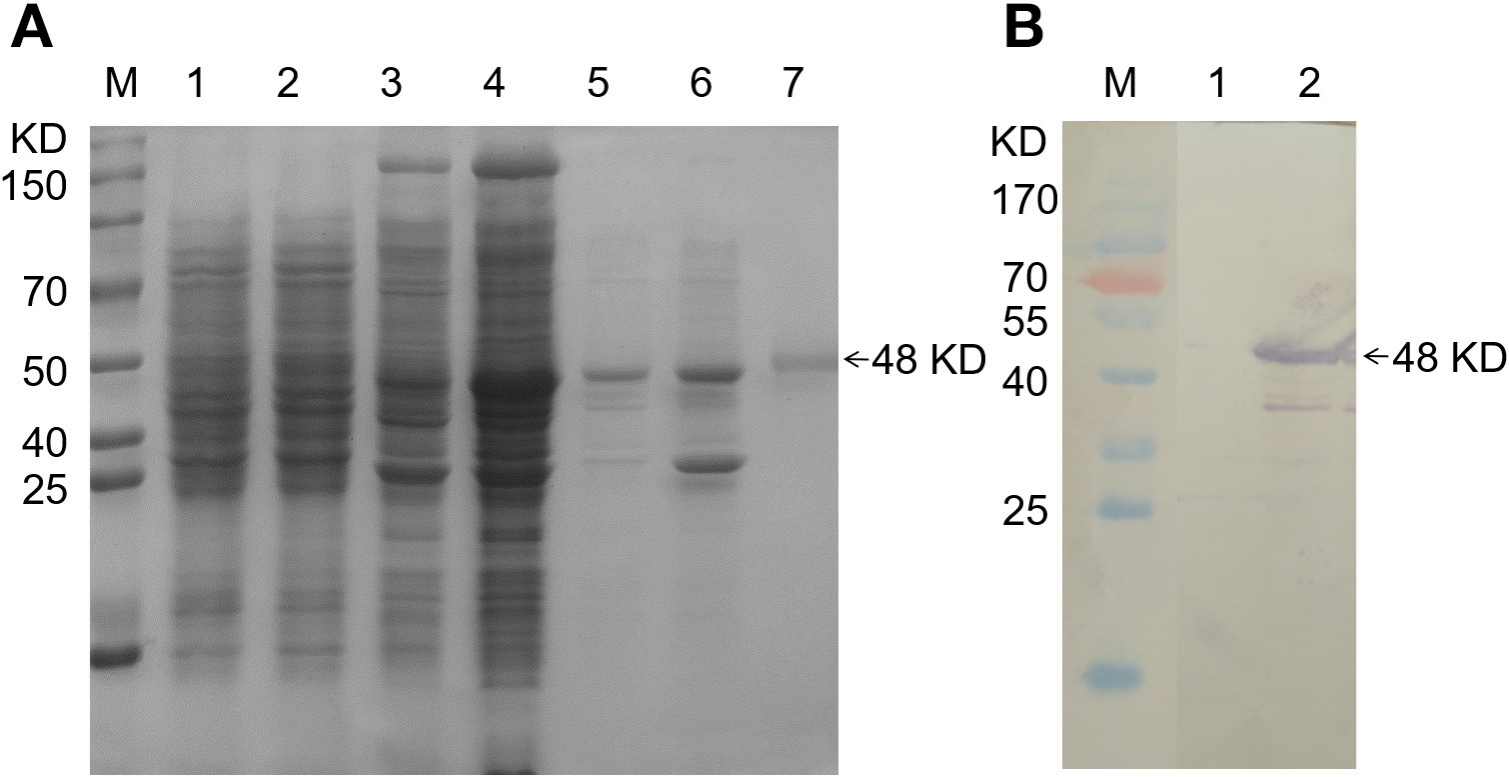
Expression and purification of pGEX-4T-1/TFG (194) protein. **(A)** SDS-PAGE analysis of the recombinant TFG (194) protein. M: Protein molecular weight Marker; lane 1, lane 3: Expression product of pGEX-4T-1 and pGEX-4T-1/TFG (194) before induction, respectively; lane 2, lane 4: Expression product of pGEX-4T-1 and pGEX-4T-1/TFG (194) of after induction, respectively; lane 5: Supernatant of expression product of recombinant TFG (194) protein after IPTG induction; lane 6: Precipitation of expression product of recombinant TFG (194) protein after IPTG induction; lane 7: Purified TFG (194) protein. **(B)** Western blot analysis of recombinant TFG (194) protein. M: Protein molecular weight Marker; lane 1: Reaction of anti-GST antibody with the bacteria lysate of pGEX-4T-1/TFG (194) before induction; lane 2: Reaction of anti-GST antibody with the purified fusion protein.

After IPTG-induced expression of the pGEX-4T-1/TFG (194)-positive plasmid, SDS-PAGE analysis showed that there was a major band at ~48 kD, which was basically consistent with the expected size of the fusion protein (Figure 2A). However, neither pGEX-4T-1 nor pGEX-4T-1/TFG (194) had corresponding bands before induction, indicating that expression of the fusion protein was successfully induced. Then, the fusion protein pGEX-4T-1/TFG (194) was purified by the gel recovery method, and Western blotting was used to verify the results with an anti-GST tag antibody. A single band of the desired size appeared (Figure 2B), which indicated that the fusion protein was purified successfully, and it can be used in the preparation of polyclonal antibodies.

**Figure 3:**
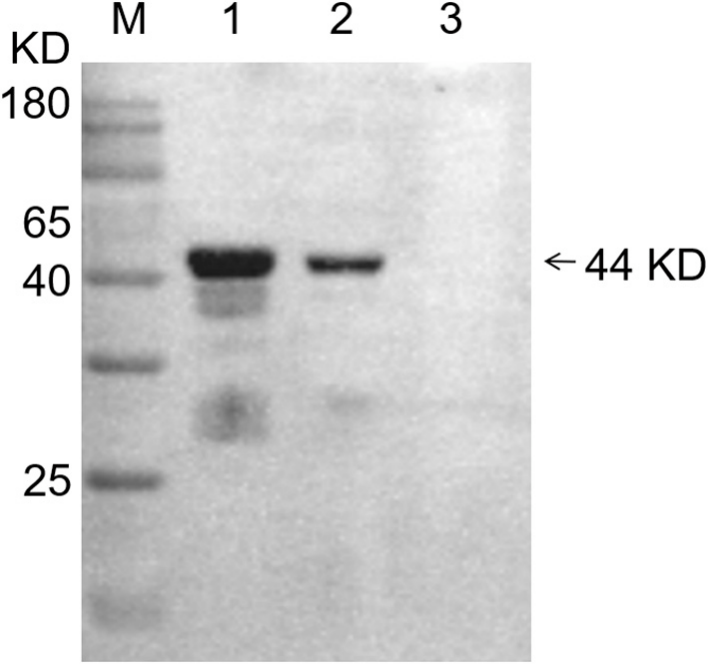
Western blot analysis of specificity of TFG (194) protein polyclonal antibodies with 3 different antigens. M: Protein molecular weight marker; lane 1: Bovine PBMCs; lane 2: The lysate of *T. annulata* schizont-transformed cells; lane 3: *T. annulata* schizont.

After antibody preparation was completed, the titer of antiserum was higher than 1:12,800, as shown by indirect ELISA detection. In addition, Western blot identification was carried out using the lysate of *T. annulata* schizont-transformed cells, bovine PBMCs and *T. annulata* schizont as antigen, rabbit immune serum as primary antibody, and HRP-conjugated donkey anti-rabbit IgG as secondary antibody. The results showed that there was a band at 44 kD, consistent with the expected size (Figure 3), indicating that the polyclonal antibody could react with the lysate of *T. annulata* schizont-transformed cells and bovine PBMCs and showed high specificity. However, TFG antibody could not react with *T. annulata* schizont.

### Effect of BW720c on the growth state of schizont-transformed cells

**Figure 4:**
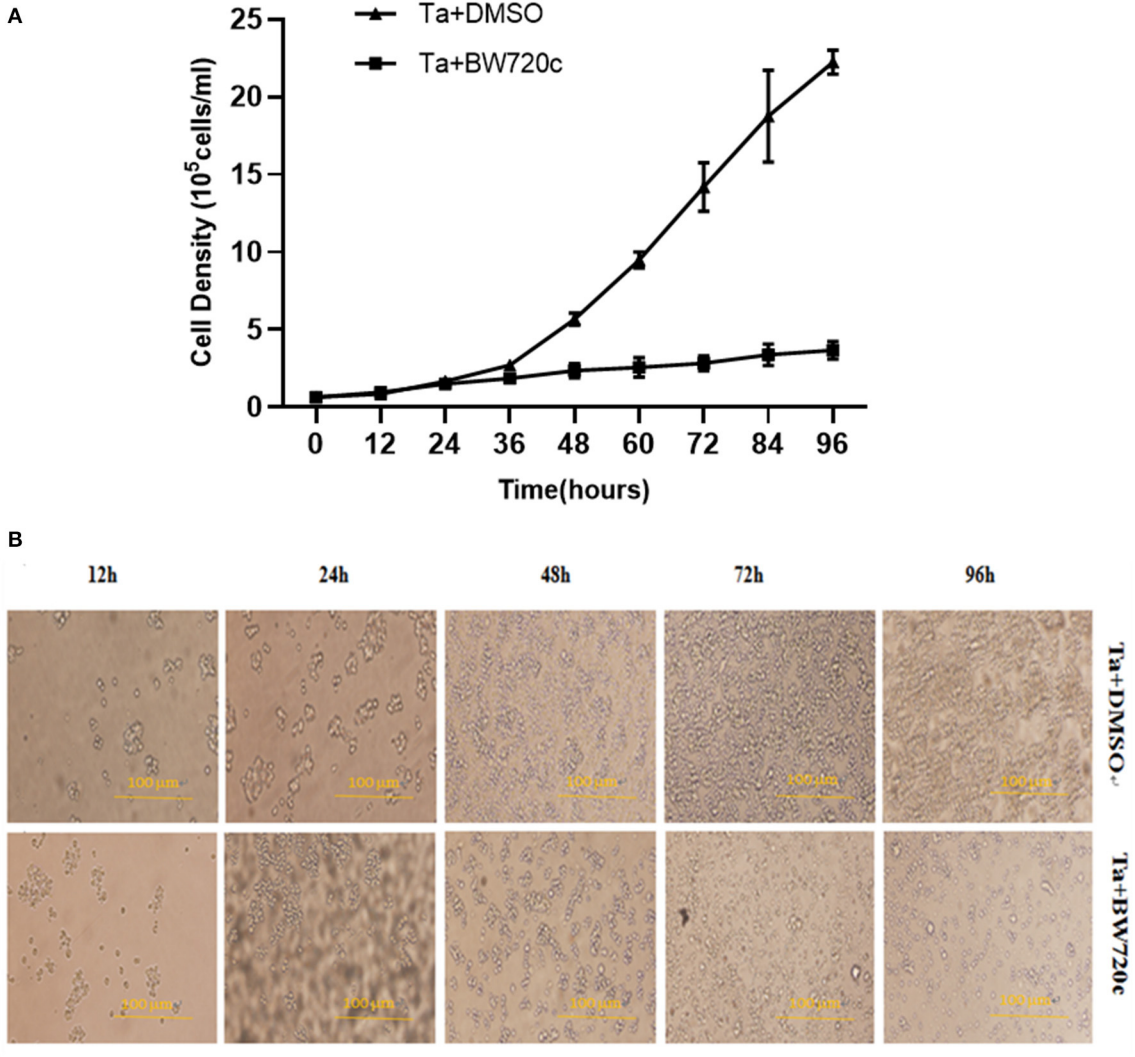
Effect of DMSO and BW720c on the growth state of *T. annulata* transformed cells. **(A)** Growth curve of *T. annulata* transformed cells treated by DMSO and BW720c. **(B)** Affects of DMSO and BW720c for proliferation of the *T. annulata* transformed cells.

By counting the number of living cells at each time point with trypan blue staining, we obtained the cell growth curve of *T. annulata* schizont-transformed cells after BW720c treatment. The results showed that the number of test group cells did not change significantly within 24 h compared with that of the control group, and the cell growth rate was substantially lower than that of the control group from 36 to 96 h. At 96 h, the number of cells in the two groups differed the most (Figure 4A). The results suggested that BW720c had an obvious inhibitory effect on the proliferation of *T. annulata*-transformed cells, and this study laid a foundation for further research on tumor-related genes.

In addition, cell morphology was observed at 12, 24, 48, 72, and 96 h by cell microscopy. The cell growth rate of the test group was slower than that of the control group, which indicated that the proliferation of the transformed cells could be effectively inhibited by treatment with this drug (Figure 4B).

### Real-time PCR and Western blotting analysis of the TFG (194) gene

**Figure 5:**
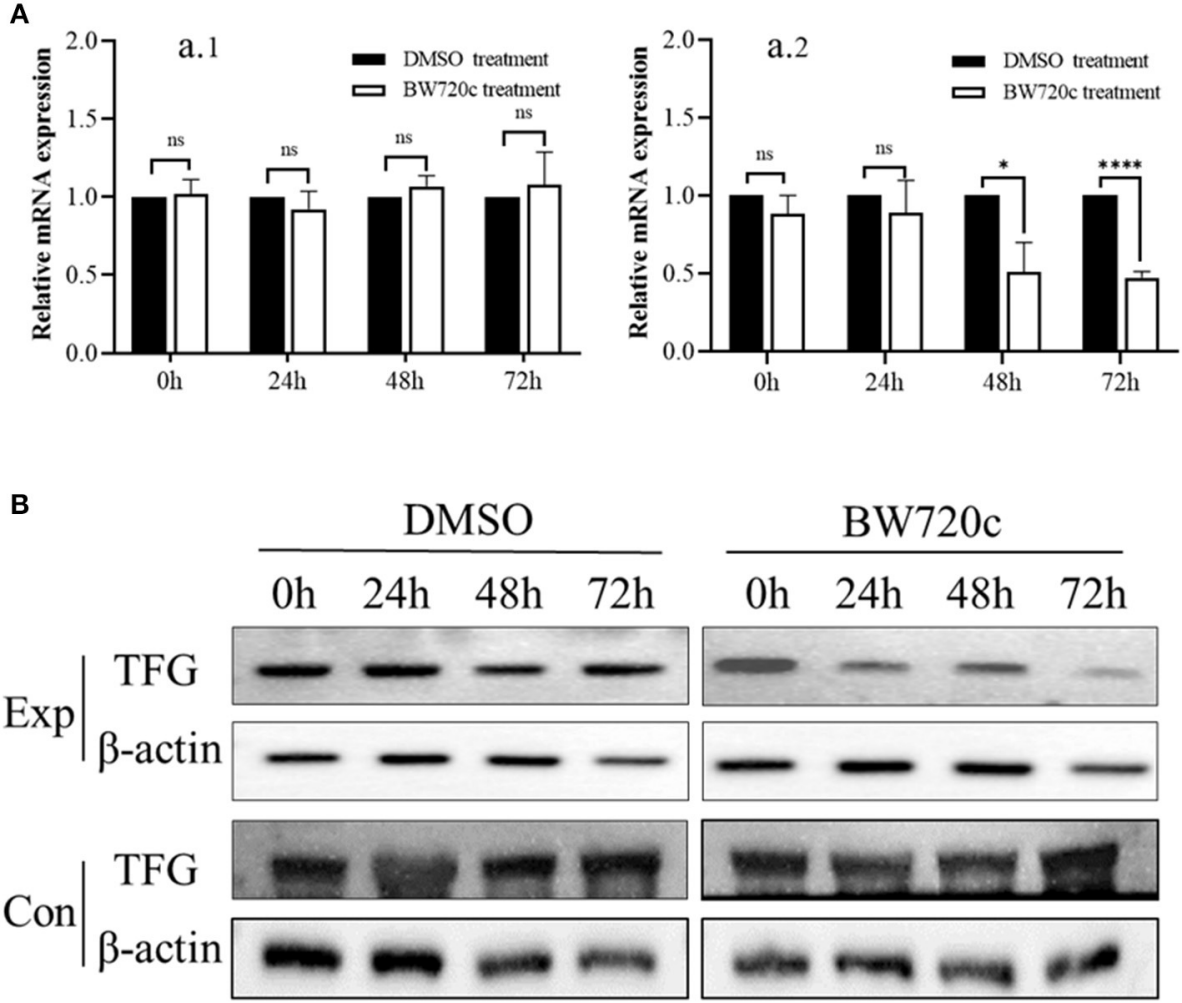
Real-time PCR and Western blotting analysis of TFG (194) on *T. annulata* transformed cell and PBMCs. **(A)** Analysis of RT-qPCR of TFG (194) gene. **(A)** 1: Each bar represents the fold change of TFG (194) gene in treatment groups (BW720c) compared with control groups (DMSO) from PBMCs of uninfected bovine. **(A)** 2: Each bar represents the fold change of TFG (194) gene in treatment groups (BW720c) compared with control groups (DMSO) from *T. annulata*-transformed cells. The gene expression level was nomalized to GAPDH of bovine and the expression level of control groups was used as calibrator to calculate the fold change. Fold change was calculated based on a mean of three biological replicates. Asterisks indicate statistically significant differences between the treatment groups and the control, using Student's *t*-test; ns, not significant (*p* > 0.05), *p < 0.05 and ****p < 0.0001. Bars on top of the histograms indicate standard errors. **(B)** Western blotting analysis of TFG(194) protein. TFG (194) protein levels was detected in treatment groups (BW720c) and control groups (DMSO). Exp: *T. annulata* transformation cells. Con: PBMCs of uninfected bovine. Mouse anti β-actin monoclonal antibody was used as the loading control.

Real-time fluorescence quantitative PCR analysis was performed on cell samples treated with drugs for 0, 24, 48, and 72 h. The results showed that, with the increase in drug treatment time, the transcription level of the TFG gene decreased in the test group (*T. annulata* transformation cells) compared with the control group (PBMCs of uninfected bovine). Therefore, we speculated that the change in the host tumor-related gene TFG in the transformed cells might be caused by *T. annulata* and that the TFG gene is likely to play an important role in cell transformation (Figure 5A).

With the cell lysates of the test group and the control group as antigens, the homemade anti-bovine TFG (194) protein antibody and the commercial mouse anti-β-actin antibody were used as primary antibodies, and we verified the TFG protein level by Western blotting. The results showed that compared with that of the control group, the expression of host TFG protein decreased significantly with BW720c treatment for 72 h (Figure 5B), suggesting that the expression level of the host tumor-related protein TFG in the *T. annulata*-transformed cells is related to parasitism by *T. annulata*.

## Discussion

TFG is a tropomyosin-receptor kinase fused gene. The protein encoded by TFG is a conserved secretory protein regulator that is located at the outlet of the endoplasmic reticulum (ER) and controls substance output in the endoplasmic reticulum, which is critical for intracellular protein secretion (22). TFG is important for COPII (coat protein complex II) vesicles between the ER and ER-Golgi intermediate compartments (ERGIC), and in the absence of TFG, COPII-coated carriers become dispersed throughout the cytoplasm (29). The TFG full-length cDNA contains 1,677 bp and encodes a 400 amino acid protein, including Phox and Bem1p (PB1), a double helix (CC) domain and an enrichment domain of serine-proline-tyrosine-glycine-glutamic acid (spygq). Both Phox and Bem1p (PB1) and the double helix (CC) domain are located in the first 194 amino acids from the NH_2_ terminus (23). TFG is involved in the system that controls cell size and participates in cell apoptosis and cell proliferation (25).

TFG was initially identified as an oncogene causing thyroid cancer. In thyroid cancer, the TRK-T3 oncogene is produced at the C-terminus of neurotrophic tyrosine kinase receptor 1 (NTRK1) fused with the NH_2_ terminus encoded by the TFG sequence. The TRK-T3-fused oncogene has tyrosine kinase activity, which can lead to the transformation of NIH3T3 cells by activating the RAS-RAF-MAPK signaling pathway in mice (30–33). TFG also acts on cAMP response element binding protein (CREB), which is a transcription factor necessary for cell growth and survival. By activating CREB or TFG through RNA interference, the number of apoptotic cells in the embryonic phase can be significantly increased (31). In papillary thyroid cancers (PTCS), the new gene fusion involves exons 1–4 from the 5′ end of the TFG fused to the 3′ end of RET tyrosine kinase, leading to a TFG-RET fusion that transforms immortalized human thyroid cells in a kinase-dependent manner (34).

Studies have shown that TFG protein is highly expressed in prostate cancer cells and tissues. The expression level in prostate cancer tissues was 63.9% higher than that in normal tissues. NF-κB double luciferase reporter activity was detected, and the results showed that TFG or PiN1 had only a weak effect on the NF-κB signaling pathway, but when PiN1 and TFG were coexpressed, NF-κB transcriptional activity was significantly enhanced. Specific silencing of TFG expression in PC3 prostate cancer cells reduced cell amplification and caused premature cell failure (35). TFG can also participate in NF-κB signaling pathway activation through fusion with NEMO (NF-κB essential modulator) and TANK (TRAF-associated NF-κB activator) (24). In addition, TFG can fuse with ALK (anaplastic lymphoma receptor tyrosine kinase), TRIM25 (tripartite motif-containing protein 25) and NOR1 (neuron-derived orphan receptor 1), which also play an important role in cell signal transduction and antiviral signaling (22, 24, 36).

We aimed to identify the changes in TFG gene expression in bovine leucocytes transformed by *T. annulata*. The *T. annulata*-transformed cells were used as research objects. BW720c was used to kill the parasite, and then, real-time PCR and Western blotting were used to detect the transcription and expression of TFG genes. Real-time fluorescence quantitative analysis showed that as the time of drug treatment increased and the level of gene transcription decreased, the expression of the TFG gene in host cells was related to the parasitism of *T. annulata*.

It was difficult to purchase commercial anti-bovine TFG protein antibody for Western blot analysis; therefore, based on previous reports, we cloned and expressed the TFG (1–194) gene with the NH2 terminal, which has important functions, to prepare rabbit antiserum. By specific detection, the natural proteins in the *T. annulata*-transformed host cells could be effectively identified. The effect of the drug treatment on the expression of TFG protein in the host cell was detected by a homemade anti-bovine TFG (194) antibody. We observed that compared with that of the control group, the expression level of host TFG protein decreased significantly in the test group when the cells were treated with BW720c for 72 h, indicating that the expression level of TFG in transformed cells was related to the parasitism of *T. annulata*. This result is consistent with Chen's research. They found that loss of TFG-1, which is the TFG homolog in *Caenorhabditis elegans*, results in apoptotic corpses. While its overexpression is sufficient to inhibit developmentally programmed cell death (25, 37). Witte et al. identify TFG-1 interacts directly with SEC-16 and controls the export of cargoes from the endoplasmic reticulum in *Caenorhabditis elegans*. Their findings provide a mechanism, which translocations involving TFG can result in cellular transformation and oncogenesis (37). Therefore, to further investigate the mechanism of TFG gene in *T. annulata* transformed cells, the proteins interacting with TFG were obtained by the protein interaction technique from the *Theileria annulata*. And then, the mechanism of TFG and its interacting proteins on cell transformation was explored.

## Conclusions

In conclusion, we found that TFG protein expression in *T. annulata*-transformed cells is relevant to *T. annulata*. The results helped to lay the groundwork for further analysis of the molecular mechanisms of interactions between *T. annulata* and host cells.

## Data availability statement

The original contributions presented in the study are included in the article/supplementary material, further inquiries can be directed to the corresponding authors.

## Ethics statement

The animal study was reviewed and approved by the protocol used in this study and all animal handling procedures were approved by the Animal Ethics Committee of the Lanzhou Veterinary Research Institute, CAAS (Permit No. LVRIAEC-2018-001).

## Author contributions

J-xL and G-qG participated in the design of the study. H-xZ and J-lL contributed to sample collection and performed the experiments and the statistical analysis. XL contributed to manuscript writing. All authors read and approved the final manuscript.

## Funding

This research was funded by the National Natural Science Foundation of China (31760727 and 31972701) and the National Key R&D Program of China (2017YFD0501200).

## Conflict of interest

The authors declare that the research was conducted in the absence of any commercial or financial relationships that could be construed as a potential conflict of interest.

## Publisher's note

All claims expressed in this article are solely those of the authors and do not necessarily represent those of their affiliated organizations, or those of the publisher, the editors and the reviewers. Any product that may be evaluated in this article, or claim that may be made by its manufacturer, is not guaranteed or endorsed by the publisher.

## Abbreviations

AP: alkaline phosphatase
BW720c: buparvaquone
cAMP: cyclic adenosine monophosphate
DMSO: dimethyl sulfoxide
c-FLIP: cellular FLICE-like inhibitory protein
cIAP: cellular inhibitor of apoptosis protein
FBW7α: F-box/WD repeat-containing protein 7
IgG: immunoglobulin G
IPTG: isopropyl thio-β-D-galactoside
OD: optical density
PiN1: peptidyl-prolyl isomerase
TFG: Trk fused gene
TFG (194): TFG N-terminal 1-194 amino acids
TGF-β/Smad: transforming growth factor-beta (TGF-β)/Smad
XIAP: X-linked inhibitor apoptosis protein.
